# Polatuzumab vedotin plus rituximab with chemotherapy in newly diagnosed pediatric stage IV Burkitt lymphoma: case report highlighting early complete remission

**DOI:** 10.3389/fimmu.2025.1684856

**Published:** 2025-11-10

**Authors:** Yanyan Sun, Mei Deng, Chunlei Zhang, Yanli Chen, Yanli Zhang, Wenli Zuo

**Affiliations:** Department of Hematology, The Affiliated Cancer Hospital of Zhengzhou University & Henan Cancer Hospital, Zhengzhou, China

**Keywords:** Burkitt lymphoma, pediatric, polatuzumab vedotin, early complete remission, case report

## Abstract

Although pediatric Burkitt lymphoma is considered curable, some patients still have a poor prognosis. Lack of early or complete response is an adverse prognostic factor. Therefore, improving patients’ sensitivity to initial treatment and achieving complete remission early are crucial for enhancing efficacy. Herein, we report two cases of children with newly diagnosed clinical stage IV Burkitt lymphoma who achieved early complete metabolic remission after only two cycles of treatment with polatuzumab vedotin plus rituximab combined with chemotherapy. Notably, this represents the first reported application of polatuzumab vedotin in pediatric patients with newly diagnosed Burkitt lymphoma.

## Introduction

Burkitt lymphoma (BL) is the most common pediatric B-cell non-Hodgkin lymphoma (B-NHL), characterized by high aggressiveness and a tendency to invade the central nervous system (CNS), with early bone marrow metastasis (1–3). Over the past 20 years, the application of high-dose, short-course chemotherapy regimens has significantly improved the survival rate of children with BL, but some patients still have a poor prognosis. Risk factors leading to unfavorable prognosis include: higher staging, elevated lactate dehydrogenase (LDH) levels, leukemia bone marrow and CNS involvement, as well as treatment-related factors such as lack of early or complete response (4–6). Once disease progression or recurrence occurs, the survival rate is less than 30% (7). Therefore, optimizing the treatment regimen to achieve complete remission as early as possible is essential to enhance efficacy. Herein, we report two cases of children with clinical stage IV Burkitt lymphoma who achieved complete metabolic remission (CMR) after only two cycles of immunochemotherapy incorporating polatuzumab vedotin plus rituximab. This marks the first documented use of polatuzumab vedotin in the treatment of children with newly diagnosed Stage IV Burkitt lymphoma.

## Case report

### Case 1

A 13-year-old boy was presented to our hospital on July 1, 2024, with a fifteen-day history of toothache and pain in the lower back and buttocks. The patient had no significant medical history. No personal or family history of malignancies was documented. Physical examination showed positive tenderness in the lumbosacral vertebrae. Magnetic resonance imaging (MRI) of the vertebral body showed abnormal signals in the thoracolumbar and sacral vertebrae and corresponding accessory areas, accompanied by spinal cord occupancy at the T3-T5 and T11-L1 levels. Laboratory data showed that LDH was elevated (2061 U/L). Epstein-Barr virus-DNA test was negative. Positron emission tomography-computed tomography (PET-CT) showed high uptake of 18F-fluoro-2-deoxy-D-glucose (18-FDG) in multiple areas of the body distributed in the abdominal and pelvic cavity, retroperitoneal space, thorax, bilateral neck regions I and II, multiple bones, thoracolumbar spinal cord, penis, and spleen. Then, a biopsy of the tumor in the right lower abdomen’s appendix area was performed. Histopathological examination confirmed Burkitt lymphoma. The results of the immunohistochemical method (IHC) were as follows: CK (-), CD20 (+), CD79a (+), PAX5 (+), CD21 (-), CD23 (-), Bcl2 (-), Bcl6 (70%+), CD10 (+), MUM-1 (-), Ki67 (90%+), CD30 (-), CD19 (+), p53 (-), ALK (-), TDT (-), CD99 (-), c-Myc (90%+); CD3 (+), CD5 (+) in background T cells. *In situ* hybridization: EBER negative. FISH: C-MYC/IGH (+), C-MYC break-apart (+). Bone marrow (BM) examination revealed 57.80% infiltration of abnormal mature B lymphocytes. Cerebrospinal fluid (CSF) examination, including cytospin morphology and flow cytometric immunophenotyping, revealed no abnormal lymphocytes. Diagnosed with BL, leukemic phase (stage IV, R4), the patient developed rapid disease progression during diagnostic workup. This was accompanied by new-onset sensory loss below T11 vertebra, first documented on July 3, 2024.

**Figure 1:**
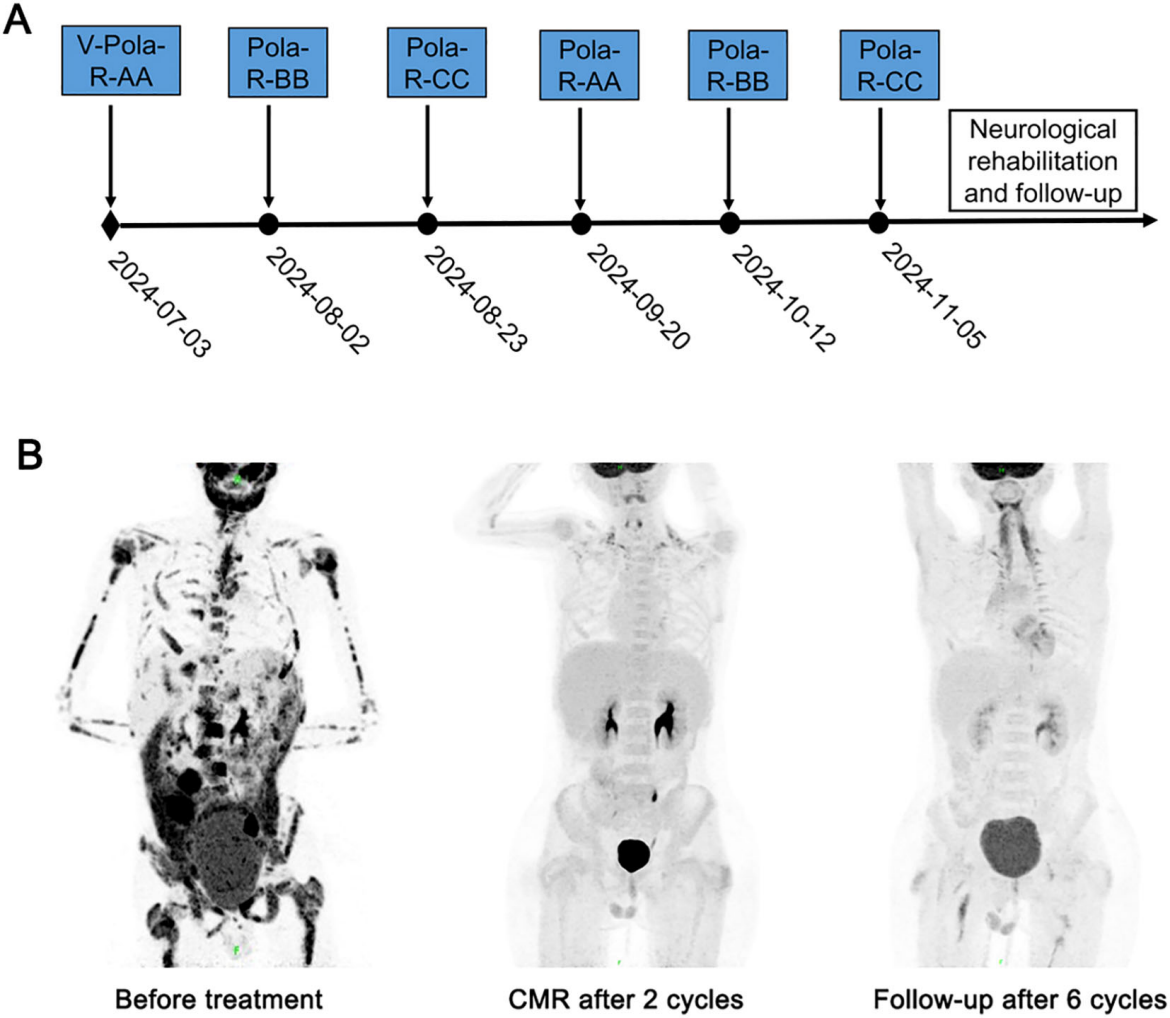
Treatment timeline and dynamic evaluation using PET-CT of case 1. **(A)** The timeline of treatment process in this case. **(B)** A dynamic evaluation of tumors with PET-CT.

The treatment was initiated after obtaining approval from the Institutional Ethics Committee and written informed consent from the guardian. Following cytoreductive pretreatment with regimen V consisted of low-dose cyclophosphamide and prednisone, a modified BFM95 immunochemotherapy regimen was initiated. This 6-cycle protocol comprised: (1) Polatuzumab vedotin plus Rituximab (Pola-R). (2) Sequentially alternating treatment blocks (AA-BB-CC-AA-BB-CC) (**Table 1**). All cycles administered at 21-day intervals and required treatment delay not exceeding 7 days for hematologic toxicity, maintaining relative dose intensity (RDI) ≥85%. During cycle 1 immunochemotherapy, the patient developed grade 4 myelosuppression complicated by febrile neutropenia (FN) and grade 2 mucositis, both resolving with supportive measures. After two cycles of immunochemotherapy, PET-CT confirmed CMR, with BM evaluation showing morphological remission. The patient completed six cycles of immunochemotherapy on November 10, 2024. Following cessation of anticancer therapy, neurological rehabilitation was initiated at the Department of Neurorehabilitation. Showing remarkable symptomatic improvement, the patient had significant recovery of sensory function below the T11 level and complete resolution of his initial back pain and toothache. Bimonthly follow-up visits showed no evidence of disease recurrence (**Figure 1**).

**Table 1:** Modified BFM-95 regimen (vincristine omitted).

Regimen	Agents	Administration	Dosage	Schedule
V	Pred	PO	30 mg/m^2^	d1-5
	CTX	ivdrip	200 mg/m^2^	d1-2
	Triple IT: MTX + Ara-C + Dex			d1
R-AA	Rituximab	ivdrip	375 mg/m^2^	d0
	Polatuzumab vedotin	ivdrip	1.8mg/kg	d1
	Dex	ivdrip	10mg/m^2^	d1-5
	IFO	ivdrip	800mg/m^2^	d1-5
	MTX	ivdrip 24h	5g/m^2^	d1
	Ara-C	ivdrip	150 mg/m^2^ q12h	d4, d5
	VP16	ivdrip	100 mg/m^2^	d4, d5
	Triple IT: MTX + Ara-C + Dex			d1, d5 (CNS+)*
R-BB	Rituximab	ivdrip	375 mg/m^2^	d0
	Polatuzumab vedotin	ivdrip	1.8mg/kg	d1
	Dex	ivdrip	10mg/m^2^	d1-5
	MTX	ivdrip 24h	5g/m^2^	d1
	CTX	ivdrip	200 mg/m^2^	d1-5
	ADR	iv	25 mg/m^2^	d4, d5
	Triple IT: MTX + Ara-C + Dex			d1, d5(CNS+)*
R-CC	Rituximab	ivdrip	375 mg/m^2^	d0
	Polatuzumab vedotin	ivdrip	1.8mg/kg	d1
	Dex	ivdrip	20mg/m^2^	d1-5
	Ara-C	ivdrip	2g/m^2^ q12h	d1, d2
	VP16	ivdrip	150 mg/m^2^	d3, d4, d5
	Triple IT: MTX + Ara-C + Dex			d1(CNS+)*, d5

Pred, prednisone; CTX, cyclophosphamide; MTX, methotrexate; Ara-C, cytarabine; Dex, dexamethasone; IFO, ifosfamide; VP-16, etoposide; ADR, doxorubicin; IT, Intrathecal injection. Pola, Polatuzumab vedotin. *For CNS-positive patients, supplement each treatment cycle with one additional triple IT.

### Case 2

A 6-year-old boy was presented to our hospital on March 14, 2025, with a 20-day history of a right neck mass. The patient’s past medical history was unremarkable. There was no personal or family history of malignancy. Physical examination revealed a palpable 6.0 × 3.0 cm mass in the right cervical region. Cervical ultrasonography revealed multiple abnormal enlarged lymph nodes in bilateral cervical regions. Laboratory data showed that LDH was elevated (2193 U/L). Epstein-Barr virus-DNA test was negative. Then, the right neck lymph node biopsy was performed. Histopathological examination confirmed Burkitt lymphoma. The results of the IHC were as follows: CK (-), CD20 (+), CD79a (+), CD3 (T+), CD5 (T+), Bcl2 (-), Bcl6 (70%+), CD10 (80%+), MUM-1 (60%+), Ki67 (90%+), CD30 (-), ALK (<1%), TDT (-), c-Myc (90%+), CyclinD1 (-). *In situ* hybridization: EBER negative. FISH: C-MYC/IGH (+), C-MYC break-apart (+). PET-CT showed high uptake of 18-FDG in multiple areas of the body distributed in bilateral cervical level II regions, right cervical level III-IV regions, left supra-adrenal soft tissue, peripancreatic area, nasopharynx, multiple bones and spleen. BM examination revealed 15.2% infiltration of abnormal mature B lymphocytes. CSF examination was negative. The patient was diagnosed with BL (stage IV, R4).

**Figure 2:**
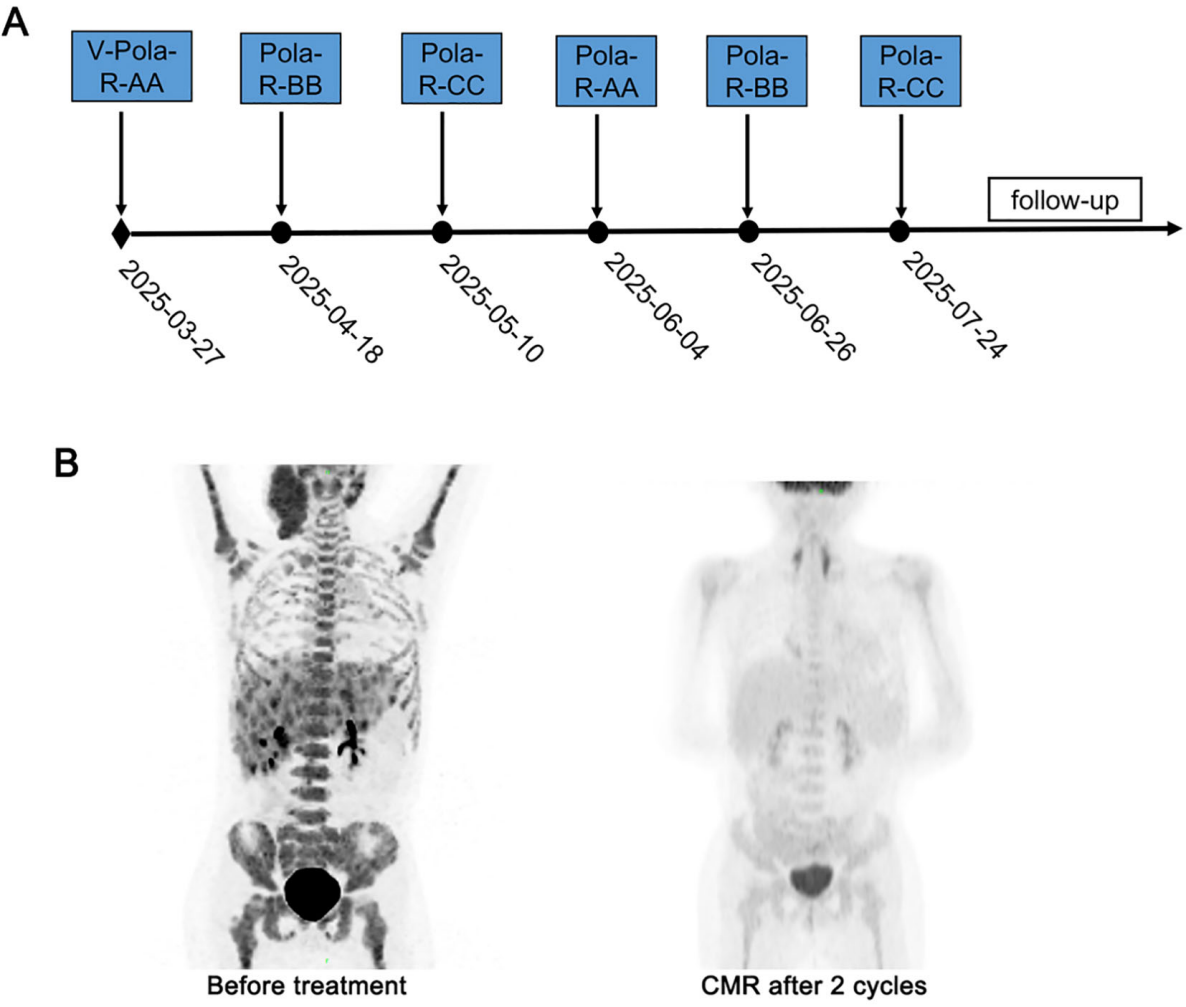
Treatment timeline and dynamic evaluation using PET-CT of case 2. **(A)** The timeline of treatment process in this case. **(B)** A dynamic evaluation of tumors with PET-CT.

After obtaining approval from the Institutional Ethics Committee and written consents from the guardian, treatment was initiated. Similar to the treatment of patient 1, this patient received immunochemotherapy based on BFM95 following cytoreductive pretreatment (**Table 1**). After two cycles of immunochemotherapy, PET-CT confirmed CMR, with bone marrow evaluation showing morphological remission. Clinically, the patient experienced rapid and complete resolution of the neck mass following the initial cycle of therapy. After the first cycle of immunochemotherapy, the patient developed grade 4 BM suppression with FN, and broad-spectrum antibiotics were administered for empirical treatment. The patient completed six cycles of immunochemotherapy on July 29, 2025 and was scheduled for follow-up similar to patient 1 (**Figure 2**).

## Discussion

In our two cases of children with clinical stage IV, high-risk Burkitt lymphoma (R4), both achieved CMR rapidly (after only two cycles of treatment) with chemotherapy combined with polatuzumab vedotin plus rituximab. The main complications were myelosuppression and mucositis, manageable with supportive care without significant treatment delays or dose reductions.

Administering risk-adapted, short-course combination chemotherapy is fundamental for BL treatment. Protocols like LMB 89/96 (French-American-British group) and BFM90/95 (Berlin-Frankfurt-Münster group) are widely recognized as standard regimens (1, 3, 8). While adding rituximab to chemotherapy has improved outcomes for pediatric high-grade B-NHL, a significant subset of high-risk BL patients fail to achieve an early complete response (CR) (9–11). Accumulating data suggest that failure to achieve early CR is associated with poorer survival outcomes and represents an adverse prognostic factor in pediatric Burkitt lymphoma (12, 13). Novel strategies to improve early CR rates are therefore crucial for this population.

Polatuzumab vedotin, an antibody-drug conjugate targeting CD79b, delivers cytotoxic drugs directly to tumor cells (14). It has shown efficacy in adult diffuse large B-cell lymphoma (DLBCL) (15, 16). Given that most BL cells highly express CD79b, polatuzumab vedotin is a rational therapeutic target. Encouragingly, polatuzumab vedotin-based regimens have achieved 100% CMR in small series of adult patients with refractory BL (17, 18). The similar CD79b expression pattern in pediatric BL suggests potential benefit.

In these two pediatric patients, we employed a regimen combining polatuzumab vedotin, rituximab, and a modified BFM95 backbone (excluding vincristine to mitigate potential neurotoxicity). Notably, both patients achieved a CMR very early in the course of therapy (after only two treatment cycles). The primary toxicities were grade 4 myelosuppression with febrile neutropenia and mucositis, which resolved with supportive care.

This report describes the first application of polatuzumab vedotin plus rituximab combined with chemotherapy in pediatric patients with newly diagnosed, stage IV, high-risk Burkitt lymphoma. Given the established poor prognosis associated with lack of early CR in high-risk pediatric BL, the rapid achievement of CMR in both cases is particularly encouraging. These promising results strongly support the initiation of prospective clinical trials investigating polatuzumab vedotin in children and adolescents with Burkitt lymphoma.

However, the study has several limitations. The findings, derived from only two patients, severely limit definitive conclusions regarding the regimen’s efficacy and generalizability. Additionally, the short follow-up duration hinders assessment of long-term outcomes—including relapse-free survival, overall survival, and potential late toxicities—associated with polatuzumab vedotin in pediatric patients. Therefore, robust evaluation of it’s efficacy, long-term safety, and survival benefit when added to standard immunochemotherapy for high-risk pediatric Burkitt lymphoma requires prospective, multi-center trials with larger cohorts and extended follow-up.

## Conclusion

Polatuzumab vedotin-rituximab-chemotherapy induced rapid complete metabolic response in two children with newly diagnosed, high-risk stage IV Burkitt lymphoma, supporting further clinical evaluation for improving early complete response rates in this population.

## Data Availability

The original contributions presented in the study are included in the article/supplementary material. Further inquiries can be directed to the corresponding author.
